# Out-of-home life spaces valued by urban older adults with limited income

**DOI:** 10.4102/ajod.v12i0.1177

**Published:** 2023-05-16

**Authors:** Hester M. van Biljon, Lana van Niekerk, Nicola A. Plastow, Lizette Swanepoel

**Affiliations:** 1 Department of Occupational Therapy, Faculty of Medicine and Health Sciences, Stellenbosch University, Cape Town, South Africa

**Keywords:** life spaces, life roles, out-of-town family, quality of life, places of worship, communities and society, public healthcare rehabilitation, medical facilities

## Abstract

**Background:**

Access to, and occupational performance in, out-of-home-life-spaces is linked to health, wellbeing and quality of life for older adults. There is little evidence of how this relates to older adults with limited resources in an African urban context.

**Objectives:**

To describe the out-of-home-life-spaces accessed and valued by older adults with limited resources, living in an urban South African setting.

**Method:**

An exploratory concurrent mixed methods study saw 84 rehabilitation clinicians conduct 393 face-to-face interviews with older adults. Clinicians produced reflective field notes and participated in focus groups. Quantitative data were analysed using descriptive statistics with SPSS Version X. Qualitative data were analysed through inductive content analysis.

**Results:**

Older adults walked, used mini-bus taxis or private vehicles to get to places of worship, medical facilities, shops, family and friends and special interest gatherings on a weekly or monthly frequency. Lack of funds was the main barrier. Older adults aspired to travel, go on holiday and to visit out-of-town family homes.

**Conclusion:**

Exploring the daily lived experience of older, urban South Africans with limited resources brought to light the value they attribute to participation in activities that contribute to the wellbeing of their families and communities. Such activities are found in a variety of life spaces.

**Contribution:**

Results could inform policy makers and service providers in their planning of community mobility, transportation services and health care, for older adults with limited resources.

## Introduction

Over the life course, people live their lives in a variety of spaces that could potentially extend from where they sleep, to places across the globe. However, as people age, they may experience life space constriction, which negatively affects their wellbeing and health (Choi et al. 2016) and reduces their quality of life (Rantakokko et al. 2016). Life space mobility includes where people go, how often they go there, and how dependent they are on others to get there (Taylor, Buchan & Van der Veer 2019). It reflects how people move in their communities, utilise community amenities, maintain social relationships and roles, and participate in meaningful activities (Rantakokko et al. 2013). Exploring older adults’ life space has value as it reflects their mobility and participation in society. A literature review (Van Biljon et al. 2022) showed that published research on the unmet community mobility needs of older adults was predominantly from higher income countries. Identifying only one source from Africa, it concluded that peer-reviewed primary research on this subject does not reflect a global drive to end discrimination, exclusion and reduce the inequalities and vulnerabilities that leave people behind (Van Biljon et al. 2022).

In older adults, there is a link between life space mobility and quality of life (Rantakokko et al. 2016). A thematic synthesis (Van Leeuwen et al. 2019) identified nine domains of older adults’ quality of life, namely, autonomy, role and activity, health perception, relationships, attitute and adaptation, emotional comfort, spirituality, home and neighbourhood and financial securtiy. Similar to the interconnectedness of key factors impacting on life-space mobility, Van Leeuwen and colleagues (2019) concluded that quality of life for older adults forms an interconnected dynamic web with the ability to access valued out-of-home places as a key component.

Morbidity, mortality and healthcare use has been associated with life space mobility (Johnson, Rodriguez & Al Snih 2020). The connection between community mobility, going to out-of-home places and the health of older adults is well documented (Margot-Cattin et al. 2019) and affected by multiple interrelated features (Franke et al. 2020). Margot-Cattin et al. (2021) found that older adults with dementia in Switzerland accessed significantly fewer places (M = 15.83) than those without dementia (M = 18.91). People with dementia were limited to visiting healthcare providers, their neighbourhood, visiting family and friends and going to restaurants or cafes. Unsworth et al.’s (2021) study of the community mobility patterns of 246 older people across seven countries found using a car as a driver or passenger, and walking were the most frequent modes of transport. Most older adults never used public transport options. Older adults who were still driving visited places like supermarkets, family and friends, and recreational facilities more often than non-drivers. Place of residence and bad weather were most likely to negatively impact out-of-home activities.

The population group interviewed in this study were learning to walk when the *Natives Affair Act* restricted the freedom of movement of black people in South Africa. When *Apartheid* was institutionalised, they were at school or trying to find work while laws restricted them from living in designated areas and dictated their right of association. They were young adults during the *decades of defiance* characterised by uprising and protests, and when Nelson Mandela was released in 1990, they were middle-aged adults. They were part of a nation that mobilised itself to vote, many of them for the first time, in South Africa’s first democratic election, showing a voter turnout percentage of 90% (SAHO20 2017). More than two decades of constitutional democracy in South Africa have seen improvements in previous discriminatory practices that affected this group’s freedom to access places and communities they value. However, the needs of older adults from low socioeconomic areas have often been overlooked (Kelly, Mrengqwa & Geffen 2019) within the gamut of South African challenges. Despite the abolition of *Apartheid* in 1994, inequity and poverty persist in South Africa and the impact that *Apartheid* had on the life spaces of a large section of older people in South Africa is still being felt. One such example is that many older people in South Africa did not learn how to drive a motor vehicle, a factor that is closely related to the types of life spaces older people can access (Huisingh et al. 2017).

In Africa, with its resource limitations, investigating engagement in out-of-home activities is important in the light of predicted pressures on transport services as urbanisation increases (Stren 2019), and the expectation that the number of persons aged 60 years and older will double between 2017 and 2050 on the continent (United Nations 2017). Sustainable solutions for healthy ageing populations in urban settings need to be addressed as a global collective to meet the challenge involved (Magnus 2012); one aspect being life space mobility. Equitable, healthy ageing within urban contexts is not a unique African challenge. Poverty has been shown to restrict independence and autonomy in old age (Gorman, Jones & Turner 2019), and low-income groups, in any given country, are most often located in peripheral locations at the edge of cities, thus experiencing transport poverty (Lucas et al. 2016).

This article reports on findings obtained in a study undertaken to explore the community mobility experiences of older adults with limited resources living in the Gauteng Province of South Africa. It reports the modes of transport they used, difficulties they experienced, strategies they employed and their suggestions for improvements. The specific focus of this article is on out-of-home-life-space-mobility, delineated to be spaces outside the boundary of participants’ own homes. The objectives were to describe the out-of-home-life-spaces accessed, report the frequency with which these were accessed, identify spaces they found difficult to go to and places they hoped to visit in future.

## Research methods and design

### The research design and process

As this was a single study collecting qualitative and quantitative data at the same time, an exploratory concurrent mixed methods study design was used. The qualitative component, which used an exploratory descriptive approach, was predominant throughout, with quantitative data complementing and confirming the qualitative findings. A 27-item semi-structured interview guide was purposefully designed to explore the community mobility and out-of-home life spaces valued by older adults who consented to participate in the research.

### Research setting

Data collection took place at eight public healthcare facilities throughout the entire Gauteng province, South Africa, that offered rehabilitation services. Public health care service users typically do not have health insurance, and many lived in peripheral townships and informal settlements (Vearey 2011).

### The research team

The research team comprised the four authors affiliated with Stellenbosch University, all with expertise in contextually relevant research in Africa, with older adults, and in the field of community mobility. Between March and May 2018, all rehabilitation clinicians working in Gauteng public healthcare facilities (N = 193) were invited to participate as field researchers in the study. A multi-professional group of 125 clinicians volunteered and attended research training workshops at their places of work and received research kits that contained the interview forms, notes, appreciation certificates and stationery needed for the research. Of these, 84 clinicians completed and complied with the research requirement. During these workshops, the ethical and methodological principles of the research were discussed and the interview form was introduced. The principles of semi-structured interviewing, keeping of field notes, professional reflection and debriefing were reviewed. The 84 clinicians were composed of 28 occupational therapists and 17 occupational therapy technicians, 21 physiotherapists and four physiotherapy technicians, 11 speech and hearing therapists and audiologists and three podiatrists. The first author kept regular contact with participating rehabilitation clinicians, coordinating data collection throughout the research process.

### Participant selection

Convenience sampling was used to recruit 393 community dwelling adults over the age of 65. Field researchers, who as per public healthcare regulation, were in uniform with their names and area of practice visible introduced themselves if there was no prior relationship. They recruited older adult participants, in-person, during participants’ routine visits to public healthcare facilities. Some participants were service users while others accompanied friends or relatives. Field researchers were asked to recruit as many participants as possible; the numbers they recruited ranged from 1 to 12, with a median of five participants. The decision to recruit older adults at primary healthcare clinics was made as these venues are strategically situated in urban areas, townships, and informal settlements and offered proficient access to the research population. All quotes in the article have participation identifiers that van be explained as follows: (1) the district in Gauteng and the number of the interview (2) the participant gender and (3) the participant age. The districts are defined by a letter: (1) T=Twane; (2) M=Johannesburg Metro; (3) E=Ekurhuleni; (4) W=West Rand; and (5) S=Sedibeng.

### Data collection and analysis

There were three components to data collection: a 27-item semi-structured interview with the participants, field notes capturing the insights and reflections of the field researchers, and focus groups facilitated by the first author with the field researchers. These focus groups captured their thoughts and insights about the interview process. This article presents an aspect from a larger study with these different sources and is focused predominantly on out-of-home life spaces emerging from the interviews with participating older adults.

The 27-item English semi-structured interview guide was paper-based and developed for the local context, based on researchers’ expertise in the field of community mobility, and their work with older adults. It was piloted in February 2018 to improve the content validity of the tool. The pilot study comprised a focus group with four older adults similar to the target population, using the speak-aloud technique (Charters 2003). Pilot participants discussed their interpretation and possible responses to each of the questions. Items shown to be misunderstood were modified and the interview guide was shortened. Requests to receive a ‘certificate’ for participation were acknowledged and included.

Semi-structured interviews, comprising 9 open and 18 closed questions, were chosen as a culturally sensitive method of data collection for the multi-ethnic study population (as opposed to a structured survey with limited response options that were pre-determined by the four white, female researchers who do not have a lived experience of the participants’ reality). The interview guide also acknowledged the importance of oral traditions in an African context (Tuwe 2016) by inviting participants to share the stories about out-of-home life spaces they valued and keeping closed question tick-off data collection to a minimum.

Data collection commenced with field researchers conducting one interview per older adult in accordance with the 27-item semi-structured interview guide. On the same day and place they went to visit a public healthcare facility, older adult participants took part in a face-to-face interview for this study. Interviews took place in rehabilitation treatment areas and were conducted by trained volunteering rehabilitation clinicians. Older adult participants came to the healthcare facilities accompanied or unaccompanied. They received a certificate of appreciation for their participation. Due to a lack of funding for the research and logistics of the research context, no refreshment was provided.

All interviews took place between 01 June and 14 September 2018. During and directly after the interviews, field researchers kept field notes in handwriting on the interview form, to capture insights and reflections they deemed noteworthy as prompted by the interview guide. Of the trained and equipped clinicians, 67% (*n* = 84) complied with all aspects of the study by conducting interviews, keeping field notes, and taking part in debriefing and discussion groups. Lastly, participating rehabilitation clinicians took part in audio recorded group discussions that were facilitated by the first author and explored final thoughts and insights; these were used to complement and enrich the findings.

The first part of the semi-structured interview captured demographical information. The second focussed on meaningful out-of-home life spaces and activities. The third captured information on participants’ mobility modes and needs, barriers they faced and strategies they employed to overcome these. In the final part of the interview, participants were asked to name the places they would go to and things they would do if they had no transport mobility restrictions. Older adults’ responses were captured by interviewing clinicians in writing on the interview forms. Additional data were provided by clinicians capturing field notes after each interview on the same form and 109 sets of reflective field notes with clinicians’ thoughts, experiences, opinions and observations were added. Concluding the data collection stage, 1 h long discussion groups at five of the clinicians’ places of work were held in October and November 2018. These cumulative 5 h of discussions were audio recorded.

The following questions from the interview guide informed the exploration of the life space mobility of participants: ‘When you leave the place you stay, your home, what are the important places you go to?’ There were six spaces in which their answers were captured under four headings that were used as prompts by the therapists. For each place named, participants were asked ‘How do you get there? How often do you go?’ (daily, weekly, monthly, yearly), ‘Why is it important for you? and Are there places you want to go to or things you want to do but find it difficulty or unable to?’ There were five spaces in which their answers were captured. For each place named, participants were asked why they did not go there, or found it difficult. ‘What places would you go to, and things would you do if you had no transport mobility restrictions?’

For the last question, interviewers prompted participants to disregard cost and health and other restrictions. For each place named, participants were asked the meaning of that place, and what mode of transport they would use to get there.

Data analysis for the results focussed on: (1) the characteristics of adults aged 65+ who attend state primary health clinics in Gauteng, South Africa, (2) the valued out-of-home places they visit, how do they get there, and why are they meaningful, and (3) where adults aged 65+ with low incomes wish they could go? The interviews, field notes and transcribed audio recordings were captured on Microsoft Excel and Word. Closed questions were captured using the response options in the interview guide. The Excel spreadsheet was uploaded to the SPSS X (IBM Corp 2020) and the qualitative data was imported to WeftQDA (Fenton 2006). A coding tree was inductively developed for the open questions on modes of transport used, and the out-of-home places. Each open question was assigned units of analysis that were added to with each subsequent interview form. A fully integrated approach, integrating qualitative and quantitative data at the data collection, data analysis, and interpretation stages was used. Data analysis and interpretation were not linear. Instead, all four authors moved back and forth between the different findings to eventually reach a consensus on the key findings.

Data analysis saw descriptive quantitative analysis of biographical data. Inductive content analysis was conducted to identify categories of out-of-home life spaces, modes of transport used and the meanings of places. Statistical analysis include counting the number of places named, the mean number of places named by each participant, the number of places visited in each category, the frequency of visiting places in each category, the number of modes of transport used and transport type for each category of place. Pivot tables and principal component analysis attempted to determine the relationship between categories of out-of-home places, frequency of visits, and modes of transport used. However, the analysis revealed no meaningful or consistent relationships.

### Trustworthiness and rigour

The face validity of the interview guide was improved by means of the pilot study. Inter-rater reliability was addressed when the first author provided the same training on data collection techniques to all rehabilitation clinicians. The operationalisation of the research was purposefully kept simple and well-documented in the protocol, consent forms and various dissemination formats to improve reproducibility of the study.

### Ethical considerations

Stellenbosch University’s Human Research Ethics Committee (HREC) provided ethical approval, Ref No: N18/01/003. Gauteng Healthcare’s Research Committee approved the research, allowing the conducting within Gauteng Public Healthcare, Ref No: DRC Ref 2018-03-008. The South African National Health Research Database (NHRD) registered the study, Ref No: GP201802 022. All work was conducted in accordance with the Declaration of Helsinki (World Medical Association 2013). Volunteering rehabilitation clinicians gave written consent as participants in this research project. They did so after orientation to the research during which they had an opportunity to question and confirm their understanding of their role in the research. Older adults gave oral consent, which was recorded on the interview form, after rehabilitation clinicians introduced the research verbally to them, informed them of their right to decline participation or withdraw with no consequences to their right to healthcare services. They were also given an information sheet of the research to take home. No demographics that could lead to identification of the older adults were captured.

Confidentiality of older adults and clinicians’ contributions were maintained throughout. All interviews were coded with only the first author having access to and ensuring safe storage of consent forms, continuing professional development (CPD) attendance registers, paper-based, electronic and audio interviews, discussion groups and feedback forms. Clinicians received CPD certificates. Other than that, they and participating older adults were not remunerated and incurred no cost as interviews were done at the healthcare facilities where they worked or that they visited for scheduled rehabilitation services. The data collection stage was concluded at participating clinicians’ places of work with a debriefing session and discussion group. In 2019, results were analysed, summarised and disseminated to all participating rehabilitation clinicians and to rehabilitation services management in the head office of Gauteng Health. This was done in the form of emails and oral presentations at staff meetings and stakeholder forums. A report was sent to the Gauteng province’s Head of Government Office.

## Results

### Participant characteristics

In total, 393 older adults, aged between 65 and 98 years (M = 72.39, s.d. = 7.17) were interviewed (see Table 1). Clinicians reported an average interview time of 10 min – 30 min per participant. Just more than half of interviewed older adults reported problems with their walking mobility (51.4%). Only 4% (*n* = 16) of the older adults lived alone. Most reported living in a brick house (91%) with indoor access to electricity and water (92.7%). Interviewed older adults shared their home with up to 18 other people (M = 3.71, s.d. = 2.31). Although more than half (52%) had no schooling or primary education, the majority spoke three or more languages (60%). Most (92.7%) were financially dependent on the non-contributory government pension and living on less than 4 US dollars a day. This income supported an average of two other people (M = 1.91, s.d. = 1.94, Range 0–12).

**TABLE 1:** Characteristics of older adults attending primary health clinics in Gauteng (*N* = 393).

Characteristic	*n*	%
**Age**	72†	Range 65–98‡
**Gender**		
Male	106	27
Female	287	73
**Citizenship**		
South African	380	97
Other African Country	12	3
Other	1	0.003
**Language proficiency**		
Monolingual	39	10
Bilingual	118	30
Multilingual	236	60
**Languages spoken**		
English	252	64
Zulu	196	50
Afrikaans	196	50
Other official South African languages	443	-
African languages outside of official South African languages	15	-
**Highest level of education**		
Never been to school	43	11
Primary school	161	41
High school	161	41
Tertiary education	28	7
**Income**		
Government old age pension§	366	92.7
Salary	15	3.8
Other	11	2.8
**Work status**		
Working	35	8.9
Never worked	20	5.1
Not Working	336	85.1
**Social capital**		
Living alone	16	4
Living with others	377	96
Type of home		
House or apartment	358	91
Zinc informal dwelling (*Mkuku)*	22	5.6
Single room	13	3.3
**Basic amenities**		
Indoor access to amenities	366	92.7
No indoor water	14	3.5
No indoor electricity	5	1.3
No indoor amenities	8	2
**Mobility**		
No problem	190	48.1
Some problem	145	37
Extreme problem	57	14.4

† , Mean;

‡ , s.d.;

§ , ±110 USD per month.

### Easy to reach out-of-home life spaces

The 393 interviewed older adults named 1118 currently visited out-of-home life spaces in total, a mean of 2.8 places per person. Inductive content analysis reduced this list to 23 categories of out-of-home places within six life space themes. These themes, in order of frequency with which they were named, included: (1) community and social spaces, (2) medical facilities, (3) shops and public amenities, (4) family and friends, (5) places for recreation and physical activity, and (6) work. All places, except annual family visits (*n* = 39), were within the urban area of Gauteng province.

#### Community and social spaces

The most named out-of-home life spaces were community and social spaces (N = 394 spaces) because ‘We must be part of our community’. Of these, places of worship were most frequently named (295/394, 75%). Places of worship offered weekly opportunities for spirituality: ‘In church people pray for me. I go for healing prayers’ (T2, Female, 65). Also: ‘I go because I love God too much’ (E6, Female, 75), and ‘So I can go to heaven when I die’ (E17, Female, 71) and ‘Because the preacher, who talks to God, tells me God loves me and this makes me feel all right’ (T19, Female, 69). Some participants were elders or deacons or fulfilled other leadership positions. Places of worship also enabled participation in charity and social upliftment activities, such as soup kitchens and support outreaches to orphanages and homeless shelters. Attending funerals, even if you did not know the deceased or the family, was reported as much valued. This involved food preparation and expression of bereavement to show support and respect towards the deceased. Some places of worship provided opportunities for older members to go camping and/or on holiday and organised festivals and events such as soccer leagues. Transport to an annual mega-church gathering where congregations gather in different provinces for a few days at a time, was also arranged. Places of worship were accessed mostly through walking (109/267, 41%) and minibus taxi (88/267, 33%) and less frequently by motor vehicle (64/267, 24%). The latter was reported to take the form of ride-share clubs.

Community support groups and special interest groups were mentioned as formal or informal gatherings taking place in private homes, funeral parlours, community halls, all-purpose community centres, healthcare centres and care homes. Seniors’ interest groups provided opportunities to ‘gym’ (exercise), grow food in communal gardens, prepare food for the soup kitchen, ‘to learn about new things’, to cook and eat together, sew and crochet, socialise, dance and sing. As one participant noted, ‘Singing in the choir is too good for my spirit’ (W12, Female, 66). Reportedly, a sewing group always began with a prayer session. Lunch was often prepared and enjoyed collectively after a group gathering, and singing and dancing occurred spontaneously, if or when the occasion allowed it. The value of the gatherings was summed by a participant as, ‘I go there to mix with people when I am lonely or stressed’ (E14, Female, 69).

Being useful and involved in helping their community was a prevalent theme throughout the interviews. An older adult reported going to the local primary school to ‘watch the children so the teachers can have tea-time’ (W9, Female, 65). Another reported ‘I visit the sick people in my community and then I pray for them’ (W29, Female, 68). Working in community vegetable gardens was noted as important for socialising and to produce ‘food to feed us’ as they also sold and processed the vegetables from the gardens for personal and community use, or to supply soup kitchens in their vicinities. At one community health centre, a retired nurse taught other older adult volunteers how to bed-wash bedridden individuals. Using designated transport, they then visited homes in their community where families required assistance with caregiving tasks. As one participant noted, ‘You are only somebody if you help somebody’ (W32, Female, 72).

Other community occupations included stokvel and burial societies (11/394, 0.03%). A stokvel is a savings scheme where by-invitation-only groups of people contribute fixed sums of money to a central fund on a weekly, fortnightly, or monthly basis. The accumulated fund is allocated to a member of the group on a rotational basis at a stokvel meeting. Burial societies, which meet regularly, are another informal financial group formed by people to cover the costs and cultural expectations of a member’s funeral. If a group member dies, all members are expected to contribute to their funeral costs.

#### Medical facilities

Although all participants were interviewed at a medical facility, only 69% (271/393) participants included these facilities in their life space. Their visits were mostly monthly (201/246, 82%). Mobility modes included walking, minibus taxi, private car, bus and other forms of transport. Participants talked about visiting medical facilities, ‘to get my medicine’, ‘because the doctor says I must go’ or because ‘I have to go to know about my sickness’. Six participants also talked about visiting medical facilities for the health needs of family members, ‘I have to take my grandchild (who is disabled) to see the therapists for his exercise’ (S35, Female, 68), and ‘My wife is in a wheelchair so I must push her to see the nurses’ (E14, Male, 70).

#### Shops and public amenities

Most participants (287/393, 73%) reported visiting shops and public amenities. These places included a variety of shopping centres, income-grant pay points, local government and postal services, and grandchildren’s school. Journeys were mostly made by minibus taxi (90/189, 47%), but also walking (53/189, 28%) and private motor vehicle (40/189, 21%). Most participants reported visiting shops and amenities monthly (263/393, 67%), with very few reporting daily visits (47/393, 12%). Shops were visited for a variety of reasons. Shopping centres often had points where participants went to receive the monthly old age grant. At shopping centres, they also purchased groceries and electricity for prepaid metres. They talked about paying accounts and running family errands as their adult children were working. Visits to shopping centres were noted as outings and socialising events, appearing to be a means of staying active and avoiding boredom. Window shopping, ‘buying with my eyes’ was a favoured pastime. One participant noted ‘I enjoy seeing all the people. Being able to talk to everybody and hearing what is happening’ (M21, Female, 70). Another participant reported visiting the shops for self-care ‘I have to do my nails and my hair… it makes me so happy’ (W17, Female, 71).

#### Family and friends

Some visits to family were frequent (weekly or monthly) and to people nearby, while other visits were annual and to ancestral homelands. A participant said:

‘I have to go to Swaziland to go and see my cattle. I love my family. We need to keep our family relations. The grandchildren must know who you are’ (E18, Male, 74).

Walking to friends and family close by was common, and was associated with daily (5/149, 0.03%) and weekly (45/149, 30%) visits. Minibus taxis and private cars were used to visit families across town, usually monthly (60/149, 40%). Of all the bus journeys reported, almost half were taken to visit family and friends (16/36, 44%). Annual trips to family in faraway places (39/149, 26%) were taken by bus, minibus taxi, or in a rideshare with someone who had a motor vehicle.

#### Places for recreation and physical activity

Places for leisure and physical activity were seldom named (*n* = 62 places). Yoga, meditation, tennis, squash, fishing and soccer (football) were named as sports. Only eight participants named theatre or cinema. Most lived in Laudium, a suburb with a predominantly Indian community. Participants talked about watching ‘our movies’ (Bollywood) in a community centre. Two participants reported visiting a beerhall (Shebeen), while one reported gambling.

#### Work

Fifteen participants reported travelling to work on a daily (12/15, 80%) or weekly (3/15, 20%) basis. They either walked (6/15, 40%), used a minibus taxi (5/15, 33%), or a private vehicle (4/15, 27%).

### Difficult to reach out-of-home life spaces

Most participants experienced restricted life space mobility, with 68% (*n* = 268) reporting 367 places they wanted to go to but found difficult to get to. Community and social spaces that were difficult to reach included political, religious and cultural gatherings. Large specialist teaching hospitals were difficult to reach medical facilities (10/268, 0.4%). Shops and public amenities that were difficult to reach included wholesale and trendy shopping outlets (69/268, 26%). Reaching family and friends was a barrier for many (179/268, 67%), as were recreational and leisure facilities (17/268, 0.6%) such as Carnival City, sports stadiums and the Johannesburg Zoo. Fifty participants reported not being able to go on holidays. Barriers were predominantly related to financial constraints (182/268, 68%), health restriction (118/268, 44%) and transport problems (35/268, 13%). Crime and community unrest (15/268, 6%) on route to, or at places of value was also a barrier. These are expanded on in Table 2.

**TABLE 2:** Barriers to visiting out-of-home places.

Barrier	Participant quotes
Financial	‘To travel is expensive’. ‘There is never enough money’.
Health and Ageing	‘I move too slowly – it makes other people angry’. ‘It is too difficult for me to go anywhere because my body is so old’ ‘When I go anywhere, I have to take the wheelchair and a helper, and we all must travel and it is too expensive’.
Public transport	‘We must not go when it is time for everybody to go to work or the children go to school. There is too much pushing and rushing. We must wait or not go to that place’. ‘We have to take lots of taxis to get to the Hospital’. ‘Lots of walking to get to the taxi ranks and this is not possible for me and is also dangerous’. ‘It takes the whole day to go somewhere’.
Crime	‘If I go to places and they take a long time to see to me I have to go home in the dark and it is too dangerous’. ‘I cannot leave the house because they break into it when they see you are not home’.
Weather	‘The weather is a big problem if it is cold or wet, I do not go’. ‘If it is cold or wet my bones cannot move. In winter I am struggling’.
Carer responsibilities	‘I have to look after my grandson so I cannot go’. ‘I take care of my wife without me she is too disabled’.

### Out of home and out of reach

Interviews were concluded with a question: What places would you go to, and things would you do if there were no restrictions or barriers that kept you from doing so? From this question, holidays emerged as an out-of-home place that was out of reach. Of all the participants who responded to this question (149/393, 38%), all wanted to travel beyond their urban area or go on holiday (*n* = 149, 100%), with destinations inside South Africa being the most popular. Many participants mentioned never having seen the ocean and wanting to go on a ‘beach holiday’. Popular tourist and historical sites were also mentioned, such as Robben Island; one participant said, ‘I want to see for my own eyes where (Nelson) Mandela and (Walter) Sisulu were put in jail’ (M31, Male, 71). Other responses indicated places similar to those mentioned as difficult to go to. Of the 353 places named, frequently mentioned places were homes of family and friends whom they had not seen for many years (140/353, 39%). Religious and/or spiritual journeys (85/353, 24%) to places such as Mecca or Jerusalem were mentioned, as were wholesale shopping outlets (80/353, 23%), day outings to the zoo (18/353, 5%) and theme parks (19/353, 5%). Participants who had worked as domestic workers in private family homes mentioned wanting to go and visit previous employers (11/353, 3%), ‘I worked for that family for 37 years and I miss them and I want to see the children; if they are married and grown up now’ (W11, Female, 69).

## Discussion

Participants could access life spaces of value that were within close vicinity. These places gave them access to activities that offered socialising, spiritual and emotional support, self-actualising and validation opportunities. A prominent feature of the activities they participated in at these places was their altruism. They valued places where they could partake in activities that helped their families, community, or people in need. They ran errands for and took care of their families and ministered to the less fortunate by working in soup kitchens and community gardens. Conversely, the findings confirmed that the out-of-home life spaces of South African older adults with limited incomes are shaped and limited by personal financial restrictions, transport poverty and crime. While prominent policies nationally (Maswanganyi 2018; Republic of South Africa 2006) and internationally (World Health Organization [WHO] 2015) recognise the need to address transport poverty and crime and its impact on older adults, the realities reported by participants illustrate a lack of implementation to effect change.

The importance of places of worship in the life spaces of older adults was noteworthy because these places had multiple meanings and expanded the life spaces of participants by offering opportunities to travel to religious gatherings and go on outings. Similar findings were evident in a study conducted in the UK (Tomalin, Sadgrove & Summers 2019) where semi-structured interviews with black, Asian and minority ethnic persons highlighted the importance of spiritual occupations for the older population group. This study’s authors call for the recognition of the importance of religion and spirituality for people attending therapeutic services.

The frequency of visits to health facilities could be the result of recruitment for this study, which was conducted at healthcare facilities and interviews held by healthcare practitioners who advocated the attendance at rehabilitation services. A comparison with older adults from the general population might be useful. Respondents reported being within walking distances to and from healthcare facilities, which was a positive indicator for South Africans’ right of access to healthcare (South African Government 1996) and universal health coverage goals (Evans, Hsu & Boerma 2013).

Shopping centres have already been identified as places of multiple meaning and value to older adults (Bruggencate, Luijkx & Sturm 2018) as have special interest groups as life spaces of value (Masoga & Shokane 2019). Culture-specific interest groups, such as *stokvel* and burial society meetings, need to be explored in more depth for the value that they offer, especially because these forms of self-help initiatives originated in response to the problems of poverty and income insecurity in communities (Matuku & Kaseke 2014).

Fiscal, opportunity and transport poverty were the predominant barriers that restricted the older adults going to life spaces they valued. Being able to go on holiday, visit popular historical sites or return to places where they grew up were the aspirations of older adults, which, in contrast, are opportunities regularly used by more affluent South Africans.

The out-of-home life space mobility of persons was limited by resource limitations more evident in spaces further away from the family home. Out-of-home life spaces closer to home were frequently visited, predominantly through walking. Drawing on the transactional nature of occupation, the restrictions imposed by macro-level infrastructure limitations on the occupations of vulnerable groups require consideration. In spaces that were accessed mostly through walking, older adults engaged creative solutions to overcome barriers, for example, walking in groups to reduce exposure to crime. However, further away spaces were affected by infrastructure limitations at system levels that require influence by local or national-level policy makers. Advocacy for transport infrastructure that is accessible to vulnerable groups, such as older adults, is a key responsibility of service providers who understand the importance of out-of-home life space in maintaining health and quality of life; this includes occupational therapists.

### Strengths and limitations of the study

The nature of the therapeutic relationship might have impacted positively on results as clinicians reported older adults enjoyed having their undivided attention resulting in lengthy detailed interviews. Another strength is that the interview guides were specifically developed for the research population and are available from the first author. Nevertheless, clinicians reported difficulty fitting the interviews with older adults into their busy public healthcare practices. Language, cultural and educational differences between interviewing clinicians and participating older adults were reported to have been a barrier, and these may have affected the quality and content of the interviews. Interviewers reported having to translate and explain abstract and open-ended questions. The nature of the therapeutic relationship might also have impacted on results, for example, reporting going to beerhalls as life spaces of value might have been censored. There were difficulties interviewing older adults with speech and/or hearing problems reported, with no mitigation registered, as only 11 of the clinicians had speech and hearing training to address this situation. Due to the uniqueness of the South African settings, and the use of a convenience sample, external validity is limited.

Another potential limitation was balanced reporting of quantitative and qualitative findings obtained using mixed methodology to answer our research question. Such complexities associated with mixed methods have been described as a potential *recipe for disaster* (Plastow 2016). This article is not situated in a positivist paradigm, in which numbers and statistical analysis present the *truth*. We were not trying to measure older adults’ life spaces (e.g. by using the Life Space Assessment) or recruit a generalisable sample of older adults. Instead, this research was situated within an interpretivist paradigm in which the subjective reality of older adults’ life spaces was of primary interest. We wanted to understand each person’s reality based on their experience of that reality. For that reason, we deliberately placed emphasis on the inductive analysis of where older adults in Gauteng go, and interpreted these findings as life spaces that were within reach, difficult to get to, or out of reach. It did not matter how far away from a person’s home a destination was, we wanted to know if they could get there or not. We viewed participants’ quotes as critical to understanding their reality. The quantitative data was complementary to this analysis. It helped us understand how many participants went to places, the frequency with which people went there, and their most common modes of transport. This article can therefore be criticised for not analysing the quantitative data to its full, objective, potential. But can also be criticised for not using in-depth semi-structured interviews with a smaller number of participants. This study and the methodology we used may raise more questions than answers. As only the second study in Africa to address the community mobility needs of older people, the hope is that the questions raised for reviewers and readers will be a spark for future research.

## Conclusions

A report (United Nations 2017) on global ageing calls for multi-sectoral policies to ensure that older persons are able to participate actively in economic, social, cultural and political life. Limited financial means, transport poverty and crime thwart the realisation of this, for older adults who were interviewed for this study. Future research is recommended to highlight additional barriers. Transport poverty and crime are addressable if there is political will, and if communities take responsibility for the well-being of their older adults. This was demonstrated by places of worship where rideshare-clubs and designated transport allowed access to events and life spaces of value. Future research may focus on the implementation and effectiveness of similar strategies in other sectors, such as retail and healthcare. Fiscal and opportunity poverty are more complex issues, and the former is a national concern in South Africa (Ramaphosa 2019). Active travel was used as a strategy by older adults in this study, as they went to life-spaces that were within walking distance from their residences. The other strategies that older people in South Africa use to maintain access to their life spaces are another interesting avenue for investigation.

The critical importance of spiritual occupations for older South Africans with limited income is shown in this study. It also brought to light the value they attribute to participation in activities that contribute to the wellbeing of their families and communities, and that such activities are found in a variety of life spaces. Policy makers, researchers and service providers should take note of this, and incorporate social participation into their planning of community mobility, transportation services and health care for this cohort. Finally, the findings of this study taught us that older adults with limited income are an underutilised support resource in South Africa, that could be activated and mobilised to the benefit of the communities they live in.
